# Supplementary material for: 3D imaging of SARS-CoV-2 infected hamster lungs by X-ray phase contrast tomography enables drug testing
Source: Sci Rep. 2024 May 29;14:12348. doi: 10.1038/s41598-024-61746-4 (PMC11137149; doi:10.1038/s41598-024-61746-4)
Supplement: Supplementary file 1 — Supplementary Information. [file 41598_2024_61746_MOESM1_ESM.pdf]

Supplementary Document: 3D imaging of SARS-CoV-2 infected hamster lungs by X-ray phase contrast tomography enables drug testing

Jakob Reichmann[1], Clement Sarrazin[2,†], Sebastian Schmale[2], Claudia Blaurock[3], Anne Balkema-Buschmann[3], Bernhard Schmitzer[2,*], and Tim Salditt[1,*]

[1]Georg-August-University of Göttingen, Institute for X-Ray Physics, Friedrich-Hund-Platz 1, 37077 Göttingen, Germany
[2]Georg-August-University of Göttingen, Institute of Computer Science, Goldschmidtstraße 7, 37077 Göttingen, Germany
[3]Friedrich-Loeffler-Institute, Südufer 10, 17493 Greifswald - Insel Riems, Germany
[*]Corresponding authors: tsaldit@gwdg.de (imaging), schmitzer@cs.uni-goettingen.de (optimum transport analysis)
[†]Current Address: Equipe RAPSODI, Centre INRIA de l'université de Lille, F-59000 Lille, France

We here present additional graphics and tables, detailing in particular data concerning the viral load measured after sample extraction by nasal swab, for different days post infection (dpi) (Tab. 1 and Fig. 1)). Fig. 2 illustrates the re-weighting procedure of the chord length, by an example plot, and Fig. 3 illustrates the relative change in chord length along the PCA direction, as well as a classification by support vector machine (SVM). Finally, Fig. 4 presents the correlation of the PCA1 component and the lung affectation score (LAS), both for (a) the control group, and (b) the drug-treated hamsters.

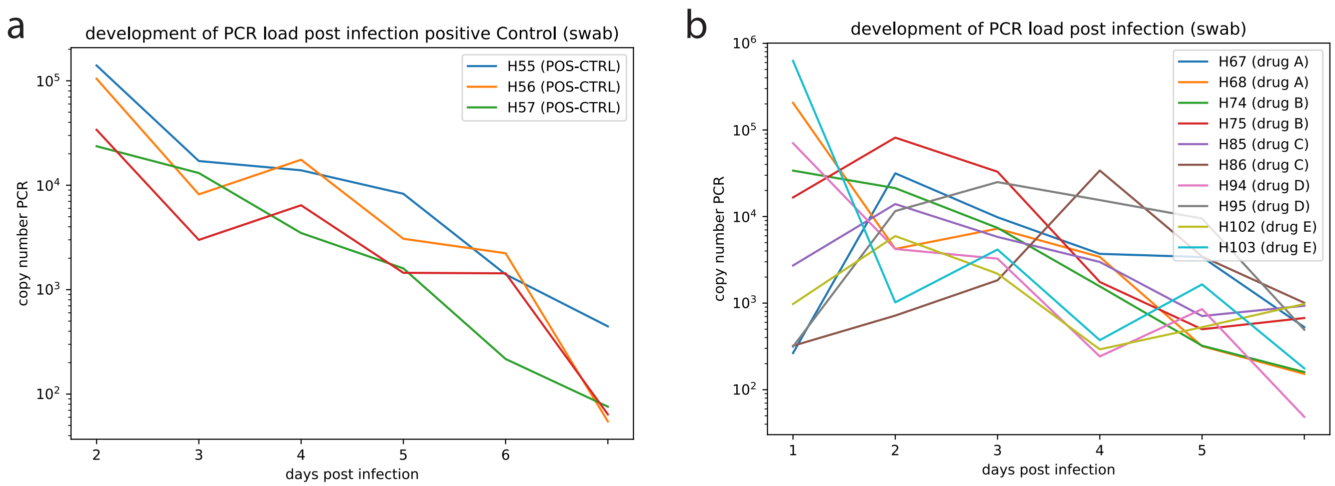

Figure 1. PCR viral load. **a** PCR copy number per μl sample as a function of day post infection (dpi) in the positive control group, and **b** in the drug-treated hamsters (selected examples only, complete data see Tab. 1). Samples were collected by nasal wash.

	0 dpi cnPCR [μl]	1 dpi cnPCR [μl]	2 dpi cnPCR [μl]	3 dpi cnPCR [μl]	4 dpi cnPCR [μl]	5 dpi cnPCR [μl]
H67	265	31570	9804	3707	3405	528
H68	205584	4231	7250	3419	318	152
H74	33918	21290	7397	1561	322	160
H75	16612	81580	32907	1754	499	673
H85	2721	13939	5820	2989	710	932
H86	321	718	1837	34015	3464	1008
H94	70295	4218	3266	242	854	49
H95	314	11570	24988	15495	9487	492
H102	977	5956	2189	293	527	979
H103	627559	1021	4160	374	1647	176

Table 1. SARS-CoV-2 gene copy number per μl sample exemplified on hamsters from different drug groups (plotted in Fig1c)

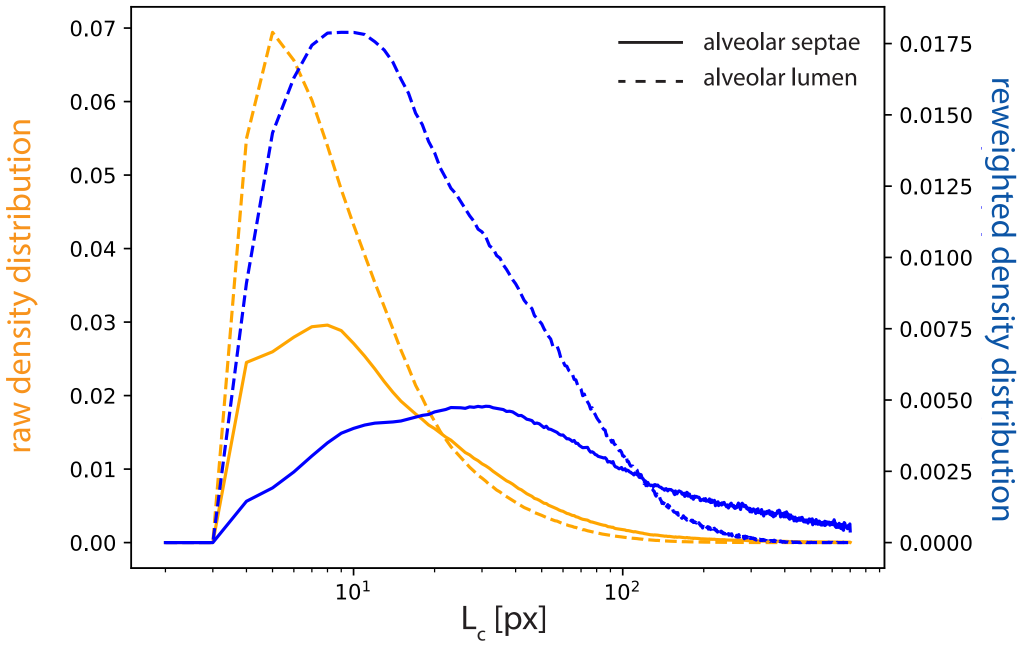

Figure 2. Illustration of reweighting. Probability distribution function (pdf) of finding chords of length L_c in septae (solid lines) and alveolar lumen (dashed lines), for the unweighted (yellow) and L_c-weighted (blue) case. Curves are shown for an exemplary POS-CTRL hamster.

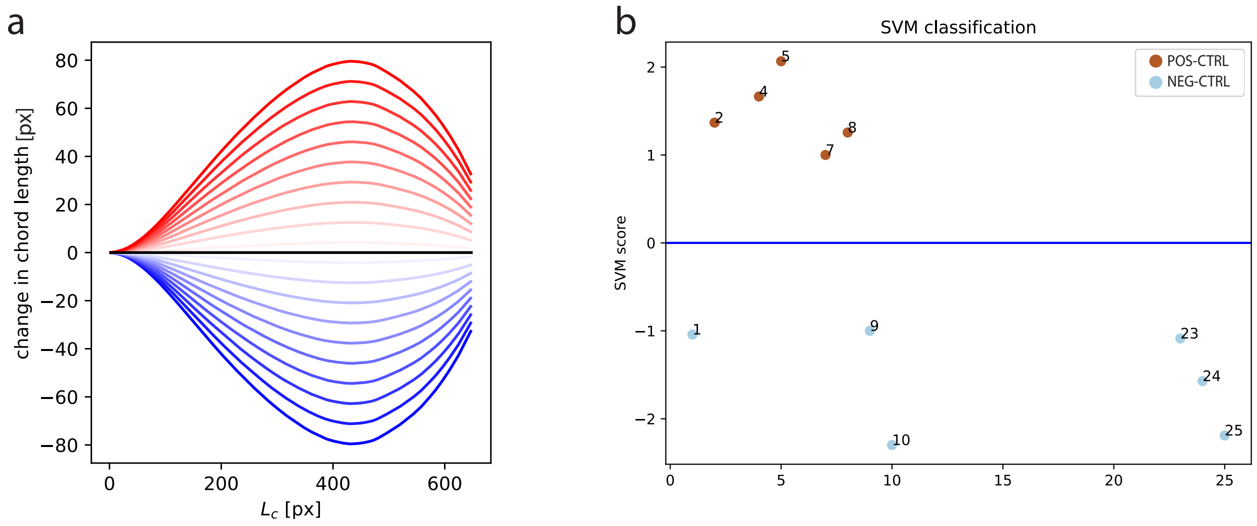

Figure 3. Relative change in CLD and SVM classification. **a** In addition to the frequency of the cords, i.e. the CLD, we can also compute the changes in chord length, when moving along PC1. The results quantify the thickening of the septae as one moves from negative (healthy) to positive (sick). **b** Distance from the hyperplane in the two-dimensional PCA space, for the control samples. The distance to the hyperplane (blue line) defines the SVM score.

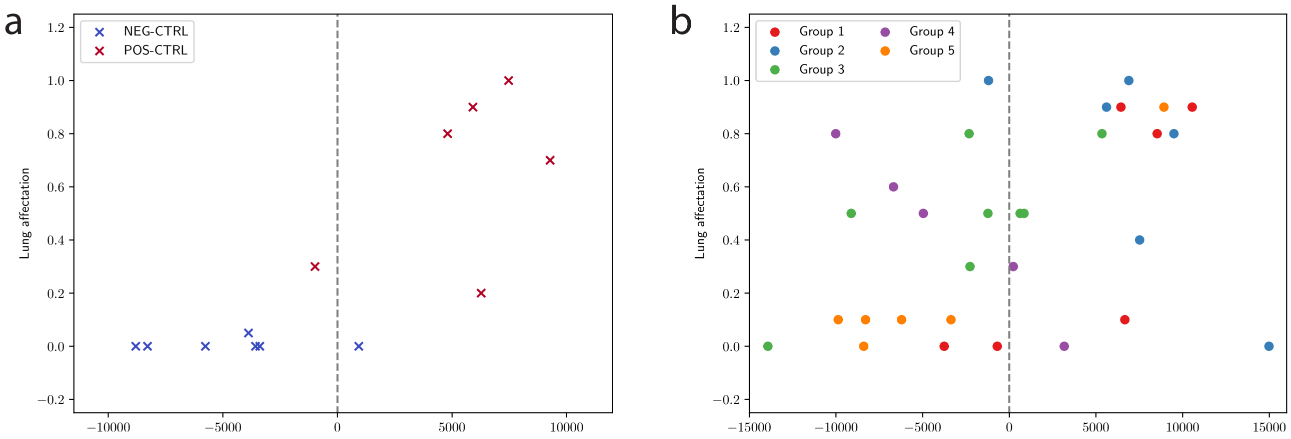

Figure 4. Representation of control (a) and drug (b) groups by their PCA 1 coordinate, plotted against their LA score. The separator at $PCA1 = 0$ is drawn as a dashed line. **a** Representation of negative (blue) and positive (red) control samples along PCA1 in the embedding space with respect to the attributed lung affectation score and the subgroups being separated by their first PCA coordinate (dashed gray line). **b** Illustration of drug groups along PCA1 in the embedding space with according to their lung affectation score.
